# Therapeutic efficacy of artemether-lumefantrine combination in the treatment of uncomplicated malaria among children under five years of age in three ecological zones in Ghana

**DOI:** 10.1186/1475-2875-11-388

**Published:** 2012-11-22

**Authors:** Benjamin Abuaku, Nancy Duah, Lydia Quaye, Neils Quashie, Kwadwo Koram

**Affiliations:** 1 Epidemiology Department, Noguchi Memorial Institute for Medical Research, College of Health Sciences, University of Ghana, P. O. Box LG581, Legon, Ghana; 2 Centre for Tropical Clinical Pharmacology and Therapeutics, University of Ghana Medical School, P. O. Box GP4236, Accra, Ghana

**Keywords:** Therapeutic efficacy, Artemether-lumefantrine, Uncomplicated malaria, Ecological zones, Ghana

## Abstract

**Background:**

In 2008, artemether - lumefantrine (AL) and dihydroartemisinin - piperaquine (DHAP) were added to artesunate - amodiaquine (AS-AQ) as first-line drugs for uncomplicated malaria in Ghana. The introduction of new drugs calls for continuous monitoring of these drugs to provide timely information on trends of their efficacy and safety to enhance timely evidence-based decision making by the National Malaria Control Programme. In this regard, the therapeutic efficacy of AL was monitored from September 2010 to April 2011 in four sentinel sites representing the three main ecological zones of the country.

**Methods:**

The study was a one-arm prospective evaluation of clinical and parasitological responses to directly observed treatment for uncomplicated malaria among children aged 6 months to 59 months using the 2009 WHO protocol for surveillance of anti-malarial drug efficacy. Children recruited into the study received weight-based 20/120 mg AL at 0, 8, 24, 36, 48, and 60 hrs. Parasitaemia levels were assessed on days 2, 3, 7, 14, 21, 28, and at any time a study child was brought to the clinic with fever.

**Results:**

A total of 175 children were enrolled into the study: 56 in the savanna zone, 78 in the forest zone and 41 in the coastal zone. Per-protocol analysis showed that the overall PCR-corrected cure rates on day 14 and day 28 were 96.5% (95% CI: 92.1, 98.6) and 95.4% (95% CI: 90.3, 98.0), respectively, with statistically significant differences between the ecological zones. The 90.4% day-28 cure rate observed in the savannah zone (95% CI: 78.2, 96.4) was significantly the lowest compared with 100% (95% CI: 93.2, 99.9) in the forest zone and 93.8% (95% CI: 77.8, 98.9) in the coastal zone (*P* = 0.017). Fever and parasite clearance were slower among children enrolled in the savannah zone. Gametocytaemia after day-3 post-treatment was rare in all the zones.

**Conclusions:**

The study has shown that AL remains efficacious in Ghana with significant ecologic zonal differences. The savannah zone may be a potential zone for any emergence of resistant alleles as a result of the slower parasite clearance observed in the zone.

## Background

Malaria remains one of the major causes of morbidity and mortality worldwide. It is estimated that in 2010 there were approximately 216 million cases of malaria worldwide, of which 81% were in Africa. In the same year, malaria accounted for approximately 655,000 deaths worldwide, of which 91% were in Africa [1]. In 2010, malaria was estimated to account for 38.2% of all out patient attendance; 34.9% of all admissions; and 33.7% of under-five mortality in Ghana [2].

The current control strategy in Ghana has case management based on prompt recognition and adequate treatment as its main focus. Chloroquine had been the first-line drug for the treatment of uncomplicated malaria in Ghana for decades until 2001, when results from studies conducted in six sentinel sites (between 1998 and 2001) showed treatment failure rates of between 8.6% and 26.8% [3]. At a consensus-building workshop in August 2003, Ghana decided to change her anti-malarial drug policy based on the evidence provided by the 2001 studies, among others. The focus of treatment options was the use of artemisinin-based combination therapy (ACT) to slow down the spread of drug resistance. This was recommended because of the rapid effect on fever and parasite clearance as well as a reduction in gametocyte carriage rates [4,5]. In January 2005, Ghana adopted the artesunate-amodiaquine (AS-AQ) combination as the first-line drug for the treatment of uncomplicated malaria [6]. Artemether-lumefantrine (AL) and dihydroartemisinin-piperaquine (DHAP) were added to AS-AQ as first-line drugs for uncomplicated malaria in Ghana in 2008 [7].

The introduction of new drugs calls for continuous monitoring of these drugs to provide timely information on trends of their efficacy and safety to enhance timely evidence-based decision making by the National Malaria Control Programme (NMCP). In this regard, a surveillance system, coordinated by the Noguchi Memorial Institute for Medical Research (NMIMR), was established in 2005 to provide data on first-line anti-malarial drugs from 10 sentinel sites across the country. This paper covers the therapeutic efficacy of AL across three ecological zones in Ghana during the period September 2010 to April 2011 using the 2009 WHO protocol for surveillance of anti-malarial drug efficacy [8].

## Methods

### Study sites

The AL study was conducted in four of the 10 sentinel sites representing the three main ecological zones of Ghana. These were Navrongo War Memorial Hospital in the savannah zone; Bekwai Municipal Hospital and Begoro Government Hospital in the forest zone; and Ewim Health Centre in the coastal zone.

The Navrongo War Memorial Hospital is located in the Kassena Nakana East district in the Upper East region of Ghana. The Kassena Nankana East district has an estimated population of 109,944 with an annual average rainfall of 950 mm. Malaria in the district is perennial with marked seasonal variation. The peak transmission season coincides with the major rains between June and October. The Bekwai Municipal Hospital is located in the Bekwai Municipality in the Ashanti region of Ghana. The Bekwai Municipality has an estimated population of 118,024. Annual rainfall in the municipality is 1,600 – 1,800 mm with double maxima rainfall in May and October each year. Malaria transmission in the Municipality is intense and perennial. The Begoro Government Hospital is located in the Fanteakwa district in the Eastern region of Ghana. The Fanteakwa district has an estimated population of 108,614. Annual rainfall in the district is 150 – 2,000 mm with double maxima rainfall in June and October each year. Malaria transmission in the district is intense and perennial. The Ewim Health Centre is located within the Cape-Coast Metropolis in the Central region of Ghana. The Cape-Coast metropolis has an estimated population of 169,894. Annual rainfall in the metropolis is 750 – 1,000 mm with double maxima rainfall in June and December each year. Malaria transmission in the metropolis is perennial [9-13].

### Study population

The study population involved all children aged between six and 59 months presenting at the Out-Patient Department (OPD) of a study site clinic with symptoms suggestive of malaria. Once a clinical diagnosis of malaria was made by the study Nurse, samples of blood were obtained from a finger prick to prepare thick and thin smears for malaria microscopy and haemoglobin level determination. Children meeting the inclusion criteria were recruited into the study and followed up for a minimum of 14 days and a maximum of 28 days. Briefly, the inclusion criteria included axillary temperature ≥ 37.5°C or history of fever during the past 24 hrs; mono-infection with *Plasmodium falciparum*; parasite count ranging between 1,000 and 250,000 per μl; haemoglobin level > 5 g/dl; absence of signs/symptoms of severe malaria; and parent’s willingness to give their consent.

Children recruited into the study received weight-based 20/120 mg AL (Coartem® – batch number x1435 supplied by WHO, Geneva) at 0, 8, 24, 36, 48, and 60 hrs. All treatments were given under direct observation by a study nurse and children were observed for 30 minutes to ascertain retention of medicine. Children who vomited during the observation period were re-treated with the same dose of medicine and observed for an additional 30 minutes. Children with repeated vomiting were given parenteral therapy with quinine as per national standard treatment guidelines and excluded from the study. All children were allowed use of antipyretics. Children who showed signs/symptoms of severe malaria, had serious adverse events or required blood transfusion were withdrawn from the study.

Outpatient follow-up visits were scheduled for days 1, 2, 3, 7, 14, 21, and 28 after treatment (day of treatment was counted as day-0). At each visit to the clinic, children were examined physically and information on symptoms, axillary temperature, and respiratory rate recorded on a Case Record Form (CRF). Parasitaemia levels (asexual and sexual) were assessed on days 2, 3, 7, 14, 21, 28, and any day within the 28-day follow-up period that a child is brought to the clinic with fever. Thick and thin smears were stained with 3% Giemsa for 30–45 minutes for quantification of asexual parasites against 200 white blood cells using a hand tally counter. Sexual parasite counts were done per 1,000 white blood cells. Parasitaemia levels were expressed per μl blood assuming white blood cell count of 8,000 per μl blood. A smear was declared negative when examination of 100 thick-film fields did not show presence of asexual parasites. For quality control purposes, all blood slides were read by two qualified independent microscopists, and discordant readings were re-examined by a third qualified independent microscopist. Discordance was defined as differences between the first and second microscopists regarding presence/absence of asexual or sexual parasites; species diagnosis; and day-0 counts meeting the inclusion criterion of 1,000 – 250,000 per μl blood. The first or second reading was taken as final depending on whichever agrees with the third reading. Filter paper blots were obtained on day-0 and at recurrence of parasitaemia for PCR genotyping, and merozoite surface proteins 1 and 2 (*msp1, msp2*), and glutamate-rich protein (*glurp*) used to distinguish between re-infection and recrudescence. Haemoglobin levels were assessed for all study children on days 0, 14, and 28.

### Data analysis

Per protocol analysis was applied in this study. Primary outcomes were treatment outcomes on day-14 and day-28 for the different ecological zones based on the WHO 2009 criteria: Early treatment failure (ETF), Late Parasitological Failure (LPF), Late Clinical Failure (LCF), and Adequate Clinical and Parasitological Response (ACPR) [8]. Secondary outcomes were patterns of fever and parasite clearance assessed for the different ecological zones using proportions (with 95% CI) of children febrile/with temperature ≥ 37.5°C or parasitaemic within the follow-up period. Haematological responses were also assessed using mean haemoglobin levels on day-14 and day-28. Proportions were compared using chi-square and Fisher’s exact tests. Normally distributed variables were compared using Student’s t-test while skewed distributions such as parasite counts were log transformed before using the normal approximation. Differences were considered significant at *p* < 0.05.

### Ethics

The Institutional Review Board of the Noguchi Memorial Institute for Medical Research, University of Ghana, reviewed and approved the study. Written informed consent was obtained from each parent/guardian at the start of the study. Each parent/guardian was informed of the objectives, methods, anticipated benefits and potential hazards of the study. They were also informed that they were at liberty to withdraw their children from the study at any time without penalty.

## Results

### Baseline characteristics

Of the 360 children screened, 175 (48.6%) met the inclusion criteria and were enrolled into the study: 56 in the savannah zone, 78 in the forest zone, and 41 in the coastal zone. Characteristics of patients at enrolment are presented in Table 1. The mean axillary temperature was significantly lowest among patients enrolled from the coastal zone. The other baseline characteristics assessed (i.e. male/female ratio, median age, geometric parasite density, and mean haemoglobin levels) did not show any significant difference between the ecological zones. There were no reports of previous anti-malarial intake prior to enrollment in all the three ecological zones. Out of the 175 patients enrolled, a cumulative total of 169 (96.6%) and 163 (93.1%) were assessed on day-14 and day-28, respectively, giving a total of six patients who were neither assessed on day-14 nor day-28. Three of these patients were lost to follow-up by day-3; 1 was withdrawn because of danger sign on day-1; 1 was withdrawn because of danger sign on day-2; and 1 withdrawn because of failure to complete treatment. The day-1 danger sign (patient from the coastal zone) was convulsion whilst the day-2 danger sign (patient from the forest zone) was excessive vomiting. Both children with danger signs were detained and given supportive treatment until they recovered.

**Table 1:** Demographic, clinical, parasitological, and haematological characteristics of patients on day of enrolment

**Characteristic**	**Total (N = 175)**	**Ecological zone**			**p-value**
		**Savannah n = 56**	**Forest n = 78**	**Coastal n = 41**	
Male/Female	85/90	30/26	39/39	16/25	0.346
Median age in months (range)	32 (6 – 59)	35 (9 – 55)	33 (6 – 59)	24 (7 – 59)	0.077
Mean axillary temperature in°C (95% CI)	38.0 (37.5, 38.4)	38.0 (37.7, 38.2)	38.5 (38.3, 38.7)	37.0 (35.1, 38.9)	0.039
GM Parasite count/μl ^a^ (range)	35009 (1240, 233608)	22775 (1240, 198440)	40983 (1423, 197080)	46669 (6647, 233608)	0.115
Mean Haemoglobin level in g/dl (95% CI)	10.0 (9.2, 10.7)	9.4 (8.9, 9.9)	10.3 (8.7, 12.0)	10.1 (9.6, 10.5)	0.557

^a^ Geometric mean parasite count.

### Primary outcomes

Out of 169 evaluable patients assessed on day-14, 163 or 96.5% (95% CI: 92.1, 98.6) showed an adequate clinical and parasitological response. Five of the six treatment failures were early treatment failures that occurred in the savannah zone. The only late parasitological failure observed on day-14 occurred in the coastal zone and was confirmed by PCR (Table 2).

**Table 2:** Treatment outcomes by ecological zone (pcr-uncorrected)

**Characteristic**	**Total (N = 175)**	**Ecological zone**		
		**Savannah n = 56**	**Forest n = 78**	**Coastal n = 41**
Classification (Day 14)				
ETF	5	5	0	0
LCF	0	0	0	0
LPF	1	0	0	1
ACPR	163	51	75	37
NA^a^	6	0	3	3
Classification (Day 28)				
ETF	5	5	0	0
LCF	5	2	0	3
LPF	10	2	3	5
ACPR	144	47	67	30
NA^a^	11	0	8	3

^a^ Not assessed.

**Figure 1:**
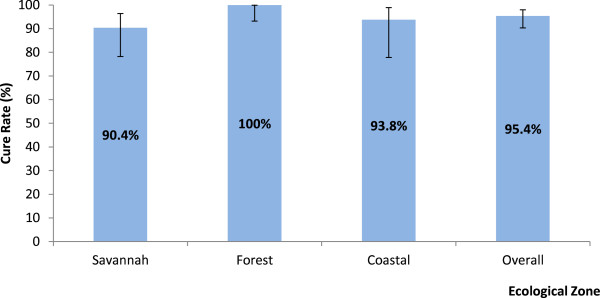
Day 28 pcr-corrected ACPR/Cure rate (including 95% CI) for evaluable patients by ecological zone.

For day-28 evaluable patients, 47 out of 56 or 83.9% (95% CI: 71.2, 92.0) in the savannah zone; 67 out of 70 or 95.7% (95% CI: 87.2, 98.9) in the forest zone; and 30 out of 38 or 79% (95% CI: 62.2, 89.9) in the coastal zone showed adequate clinical and parasitological response (Table 2). Pcr-corrected outcomes on day-28 showed an overall cure rate of 95.4% (95% CI: 90.3, 98.0). Cure rate within the savannah zone was significantly the lowest (90.4%; 95% CI: 78.2, 96.4) compared with 100% (95% CI: 93.2, 99.9) in the forest zone and 93.8% (95% CI: 77.8, 98.9) in the coastal zone (*p* = 0.017) (Figure 1).

### Secondary outcomes

**Figure 2:**
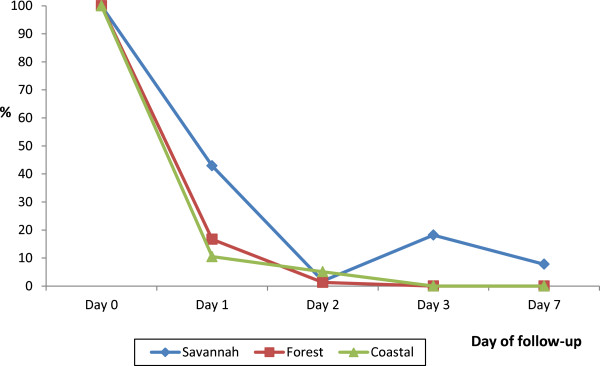
Proportion of patients with fever during first follow-up week by ecological zone.

Generally, proportion of children who were febrile during the first follow-up week decreased with day within the forest and coastal zones. Children in the two zones remained afebrile on day-3 and day-7 post-treatment. Although the proportion of febrile children in the savannah zone significantly decreased from 100% on day-0 to 1.8% (95% CI: 0.1, 10.8) on day-2, the proportion increased to 18.2% (95% CI: 9.5, 31.4) on day-3 before decreasing again to 7.8% (95% CI: 2.5, 19.8) on day-7. Febrile cases were, therefore, recorded on all days within the first follow-up week in the savannah zone. The proportion of children still febrile on day-1 post-treatment was significantly higher in the savannah zone (42.9%; 95% CI: 30.0, 56.7) compared with the forest zone (16.7%; 95% CI: 9.5, 27.2) and the coastal zone (10.5%; 95% CI: 3.4, 25.7) (*p* < 0.001) (Figure 2).

**Figure 3:**
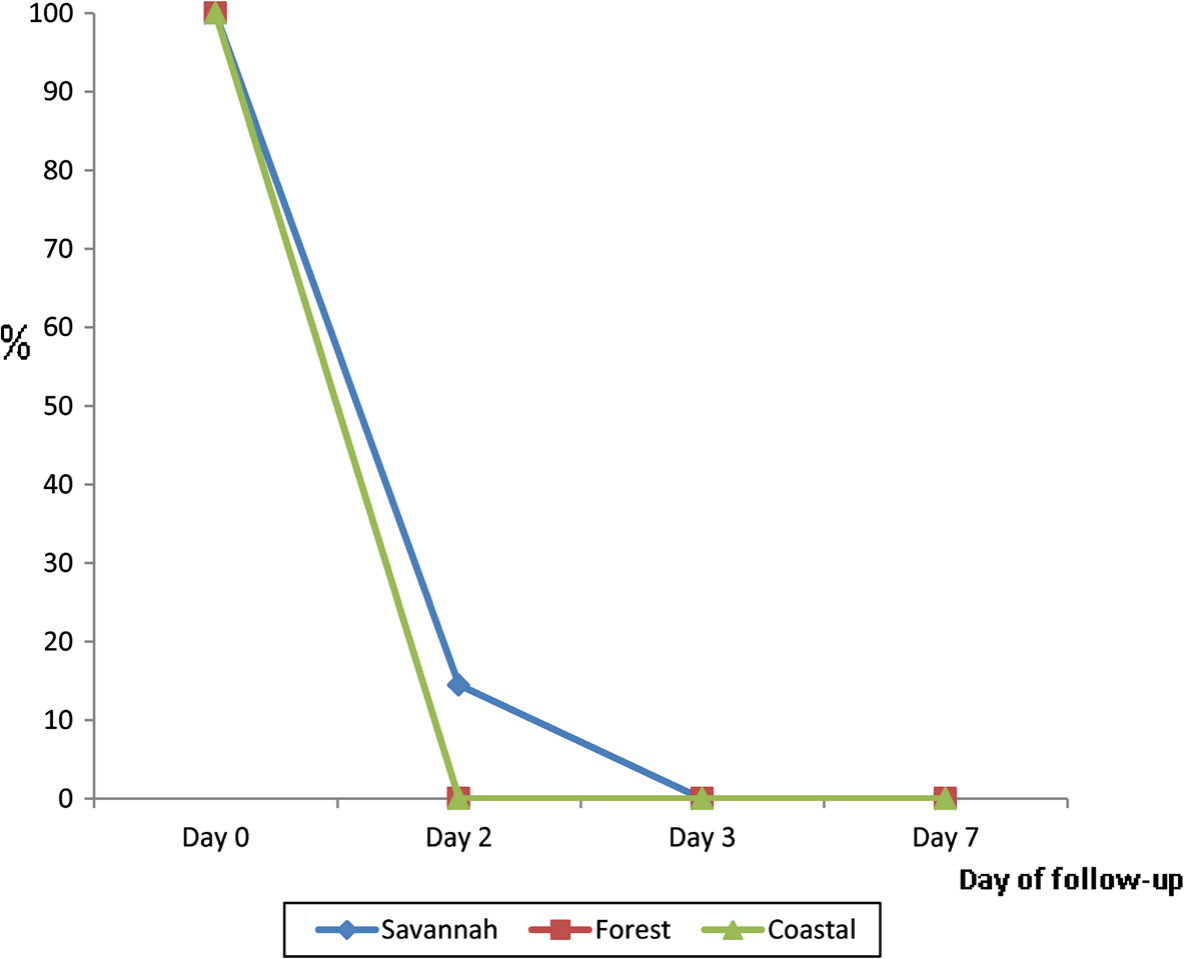
Proportion of patients with parasites during first follow-up week by ecological zone.

**Figure 4:**
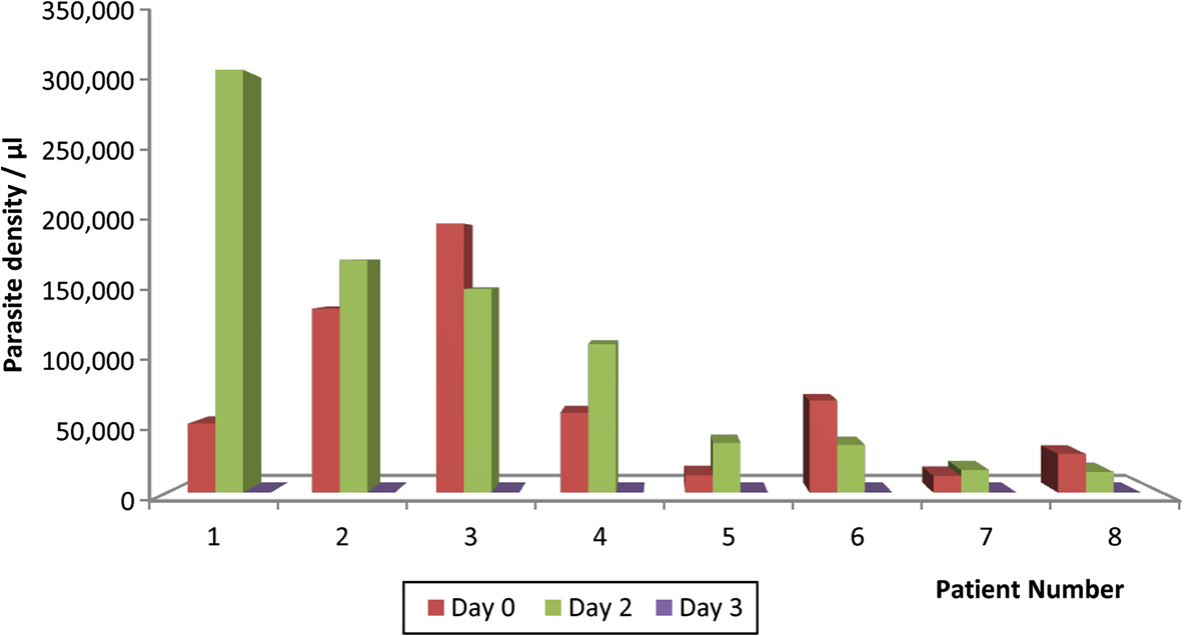
**Pattern of parasite clearance among children parasitaemic on day**-**2 in the Savannah zone.**

Regarding parasitaemia, all children within the forest and coastal zones cleared their parasites by day-2 post-treatment, and none of them was parasitaemic on day-3 and day-7. In the savannah zone, 8/55 or 14.5% (95% CI: 6.9, 27.2) were parasitaemic on day-2. However, no child was parasitaemic on day-3 and day-7 (Figure 3). A further analysis of the children who were parasitaemic on day-2 showed that 5/8 (62.5%; 95% CI: 25.9, 89.8) had parasite count greater than the day-0 count (Figure 4) and were thus classified as ETF as per protocol. All the children became aparasitaemic on day-3 without any rescue treatment to those classified as ETF (Figure 4). Parasite recurrence was observed on day-21 in one of the eight children who were parasitaemic on day-2.

**Figure 5:**
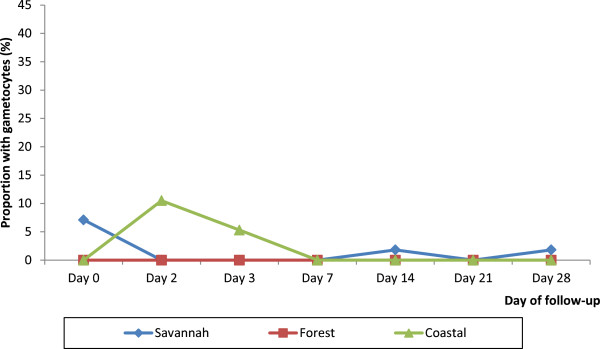
Gametocyte carriage rate during the follow-up period by ecological zone.

At enrolment, gametocytaemia was prevalent only in the savannah zone at a rate of 7.1% (4/56). By day-2 post-treatment, prevalence of gametocytaemia had declined to 0% and remained so in the zone on days 3, 7, and 21. Days 14 and 28 in the savannah zone had only 1 child (1.8%) with gametocytes. Gametocytaemia was absent on all follow-up days among children enrolled in the forest zone. For children enrolled in the coastal zone gametocytaemia was prevalent on day-2 post-treatment at a rate of 10.5% (4/38), halved by day-3 at a rate of 5.3% (2/38), declined to 0% by day-7, and remained same throughout the remaining follow-up days (Figure 5).

There were no significant differences between mean haemoglobin concentration assessed prior to treatment on day-0 and mean haemoglobin concentration assessed on day-14 and day-28 post-treatment among children in the savannah and forest zones. For children in the coastal zone, mean haemoglobin concentration significantly increased from 10.1 g/dl (95% CI: 9.6, 10.5) on day-0 to 11.0 g/dl (95% CI: 10.6, 11.4) on day-28 (*p* = 0.004) (Table 3).

**Table 3:** Changes in mean haemoglobin levels by ecological zone

	**Ecological zone**		
	**Savannah**	**Forest**	**Coastal**
Day 0 Mean Hb (95% CI)	9.4 (8.9, 9.9)	10.3 (8.7, 12.0)	10.1 (9.6, 10.5)
Day 14 Mean Hb (95% CI)	9.6 (9.2, 10.0)	10.0 (9.7, 10.3)	10.6 (10.2, 11.0)
Day 28 Mean Hb (95% CI)	10.0 (9.6, 10.4)	10.6 (10.3, 10.9)	11.0 (10.6, 11.4)

## Discussion

Following the inclusion of artemether - lumefantrine combination as one of the first-line drugs for the treatment of uncomplicated malaria in Ghana in 2008 [7], there was the need to study its therapeutic efficacy in four sentinel sites representing the three main ecological zones of the country during the 2010/2011 surveillance period.

The study has shown that the overall PCR-corrected cure rate for Ghana is 95.4% (95% CI: 90.3, 98.0) with the savannah zone showing a significantly lower rate of 90.4% (95% CI: 78.2, 96.4). The over 90% cure rate of AL in Ghana is comparable with findings from other studies in Africa within the past decade [5,14-20], and suggests that Ghana as a whole has not reached the 10% cut-off failure rate necessary for the review of AL as one of the first line drugs in the treatment of uncomplicated malaria in the country [8]. However, the 9.6% early treatment failure rate observed in the savannah zone is worrying and suggests the need for conduct of pharmacokinetic studies during subsequent efficacy studies to determine the adequacy of plasma drug concentrations, which is necessary for describing treatment failures [21].

Fever and parasite clearance were slower among children enrolled in the savannah zone. Whereas no child was febrile on day-3 and day-7 in the forest and coastal zones, febrile cases were reported on all days among children in the savannah zone during the first follow-up week. The proportion of children still febrile on day-1 post-treatment was significantly highest in the savannah zone (42.9%; 95% CI: 30.0, 56.7) compared with the forest zone (16.7%; 95% CI: 9.5, 27.2) and the coastal zone (10.5%; 95% CI: 3.4, 25.7) (*p <* 0.001). Additionally, 8/55 or 14.5% (95% CI: 6.9, 27.2) of the children enrolled in the savannah zone were parasitaemic on day-2 post-treatment whilst no child was parasitaemic on the same day in the forest and coastal zones. Out of the 8 parasitaemic children 5 (62.5%; 95% CI: 25.9, 89.8) had parasite count greater than the day-0 count but remained aparasitaemic on days 3, 7, 14, 21, and 28 post-treatment without any rescue treatment. The rapid rise in asexual parasitaemia after commencement of ACT treatment has been reported in Nigeria and attributed to a possible larger load of sequestered parasites in deep tissues in some patients. In the Nigeria study, parasitaemia levels peaked at one hour after the first dose of AL, and by 16 hours post-treatment all parasites had been cleared among children with initial rise in parasitaemia [22]. In our study, total asexual parasite clearance for children with increased parasitaemia on day-2 was achieved on day-3. This observation, coupled with the known phenomenon of parasite mobilization from deep tissues by ACT raises concern about the WHO criteria for ETF classification in relation to parasite densities [23]. It may be worthwhile shifting the criteria from day-2 to day-3 post-treatment in areas of intense transmission. This notwithstanding, the delayed clearance of fever and parasites in the savannah zone need to be further studied taking into account the possible effects of ACT usage on parasite dynamics within the population [24].

Pre-treatment gametocytaemia was prevalent only in the savannah zone (7.1%) and declined to 0% by day-2 post-treatment whilst gametocytaemia was prevalent on day-2 post-treatment in the coastal zone (10.5%), halved by day-3 (5.3%), and declined to 0% by day-7 post-treatment. This finding suggests that ACT remains efficacious in gametocyte clearance in Ghana. The efficacy in gametocyte clearance has the advantage of potentially slowing down the transmission of resistant alleles [25].

Post-treatment mean haemoglobin concentration was significantly higher than pre-treatment concentration in the coastal zone but not the forest and savannah zones. This finding suggests that the therapeutic efficacy of AL resulting from rapid parasite clearance may not necessarily translate into significant post-treatment increases in haemoglobin concentrations during a 28-day follow up period. A couple of studies in Africa have reported non-significant post-treatment increases in haemoglobin concentrations with AL treatment during a 28-day follow-up period of patients treated for acute uncomplicated malaria [15,26] whilst a couple of studies in Ghana have reported significant increases during the same follow-up period [5,27].

## Conclusions

There is evidence to show that AL remains efficacious in Ghana with significant ecologic zonal differences. The savannah zone may be a potential zone for any emergence of resistant alleles as a result of the slower parasite clearance observed in the zone. Pharmacokinetic studies will be useful in future anti-malarial drug resistance surveillance activities in Ghana to better describe treatment failures. Ghana’s policy of multiple first-line therapy is in the right direction, and needs to be supported by all stakeholders as it delays the emergence and slows spread of drug resistance [28,29]. Furthermore, the WHO criteria for ETF may need to be reviewed following the observation that all 5 children classified as ETF on the basis of parasite count on day-2 being greater than parasite count on day-0 cleared all parasites on day-3, without any rescue treatment, and remained aparasitaemic during the follow-up period.

## Competing interests

The authors declare that they have no competing interests.

## Authors’ contributions

BA and KK conceived and designed the study. ND and NQ carried out molecular genetic studies to distinguish between re-infection and recrudescence. BA and LQ coordinated the study. BA and KK participated in the data analysis. BA drafted the manuscript. All authors read and approved the final manuscript.
